# Expression and purification of recombinant proteins based on human prostate stem cell antigen and heat shock protein-70

**DOI:** 10.3892/etm.2013.967

**Published:** 2013-02-20

**Authors:** LEI DONG, XIAOPENG ZHANG, CHANGMING YU, JUN REN, LIHUA HOU, LING FU, SHAOQIONG YI, WEI CHEN

**Affiliations:** 1 Clinical Laboratory Center, PLA Air Force General Hospital, Haidian, Beijing 100142, P.R. China; 2 Beijing Institute of Biotechnology, Academy of Military Medical Sciences, Fengtai, Beijing 100071, P.R. China

**Keywords:** prostate stem cell antigen, heat shock protein, structural domain, recombinant fusion protein

## Abstract

The aim of this study was to express and purify recombinant proteins based on human prostate stem cell antigen (PSCA) and heat shock protein-70 (HSP70). The PSCA gene and various structural domains of HSP70 were amplified by polymerase chain reaction (PCR) with the respective primers. Then, the PSCA was cloned into the prokaryotic expression vector pET21a(+) with the amino-terminus, carboxyl-terminus and overall length of HSP70, by enzyme digestion to construct the recombinant plasmids pET21-PSCA-HSPN, pET21-PSCA-HSPC and pET21-PSCA-HSP, respectively. After being expressed in *Escherichia coli (E. coli)* by isopropyl β-D-1-thiogalactopyranoside (IPTG) induction, recombinant fusion proteins were purified. Western blotting was performed to confirm the expression of the recombinant proteins. The results revealed that recombinant plasmids were successfully constructed. The PSCA-HSPC and PSCA-HSP expressed in *E. coli* existed in soluble form, as confirmed by sodium dodecyl sulfate-polyacrylamide gel electrophoresis (SDS-PAGE). The purity of the recombinant proteins PSCA-HSPC and PSCA-HSP reached >95% following purification with the nickel-nitrilotriacetic acid (Ni-NTA) resin, Phenyl-Sepharose Fast Flow and Superdex 75, which lays a foundation for the development of vaccines for prostate cancer.

## Introduction

Prostate cancer is the most common non-cutaneous cancer with a high mortality rate in American males. Previously, studies on novel anticancer strategies have introduced prostate cancer immunotherapy, which represents a highly attractive therapeutic strategy for cancer treatment. Prostate stem cell antigen (PSCA), first described by Reiter *et al*, is a surface glycoprotein and has a 30% homology with stem cell antigen-2 (1). PSCA is expressed in 85% of prostate cancer specimens with high tissue specificity. There is a direct correlation between the expression level of PSCA and tumor stage, grade and bone metastasis (2). To date, studies have confirmed that vaccination based on PSCA enhances the cellular and humoral immune responses and inhibits the growth of PSCA-expressing tumors in mice (3–5). Therefore, PSCA may be a potential target for prostate cancer immunotherapy.

Heat shock protein-70 (HSP70) is a major molecular chaperone, which assists in transport, folding and assembly of proteins in the cytoplasm transmembrane. It is also involved in the functions of the mitochondria, endoplasmic reticulum and nucleus. Previous studies have demonstrated that vaccination with HSP70-peptide complexes elicit specific antitumor responses (6–9). These findings suggest that HSP70 is involved in the process of antigen presentation and has potential as a chaperone for specific antigens in vaccines.

In the present study, we successfully constructed recombinant plasmids based on PSCA and HSP70 and obtained soluble recombinant proteins with a purity >95%, which lays the foundation for the development of a vaccine for prostate cancer.

## Materials and methods

### Main reagents

The plasmids pET21a(+), pMD-HSP70 and pMD-PSCA were stored in the Beijing Institute of Biotechnology, Academy of Military Medical Sciences. *Escherichia coli (E. coli)* DH5α and BL21(DE3), pMD18-T, LA Taq, T4 DNA ligase, DNA marker 2000 and the restriction enzyme were purchased from Takara Biotechnology Co., Ltd. (Dalian, China). A plasmid mini kit was purchased from Promega Biotech Co., Ltd. (Beijing, China). Anti-PSCA polyclonal antibody and fluorescein isothiocyanate (FITC)-conjugated goat anti-rabbit IgG antibody were purchased from Santa Cruz Biotechnology, Inc. (Santa Cruz, CA, USA). Purification medium was purchased from Qiagen (Beijing, China). All other domestic reagents used in this study were of analytical grade. The study was approved by the ethics committee of the Academy of Military Medical Sciences (Beijing, China).

### Construction of expression plasmids

All constructions were cloned into the pET21a(+) vector. The human PSCA gene was amplified from the pMD-PSCA vector with the primers 5′-CCG CAT ATG TGC TAT AGC TGC AAA GCC-3′ and 5′-CCG GAA TTC CAG GGC ATG GGC CCC GCT-3′ and cloned into the *Nde*I and *Eco*RI sites of pET21a(+) to generate pET21-PSCA. The amino-terminus, carboxyl-terminus and overall length genes of HSP70 were amplified from the pMD-HSP70 vector with the primers 5′-CCG GAA TTC ATG GCC AAA GCC GCG GCG-3′ and 5′-CCG CTC GAG TCG CTT GTT CTG GCT GAT-3′, 5′-CCG GAA TTC CTG AAC AAG AGC ATC AAC-3′ and 5′-CCG CTC GAG ATC TAC CTC CTC AAT GGT-3′, 5′-CCG GAA TTC ATG GCC AAA GCC GCG GCG-3′ and 5′-CCG CTC GAG ATC TAC CTC CTC AAT GGT-3′, respectively. Then, amplification products of various structural domains of HSP70 were cloned into the *Eco*RI and *Xho*I sites of pET21-PSCA to generate pET21-PSCA-HSPN, pET21-PSCA-HSPC and pET21-PSCA-HSP. PSCA amplification was performed for 3 min at 94°C, immediately followed by 30 sec at 94°C, 30 sec at 55°C and 30 sec at 72°C for 30 cycles. HSPN, HSPC and HSP were incubated for 3 min at 94°C, followed by 30 sec at 94°C, 30 sec at 55°C and 90 sec at 72°C for 30 cycles, respectively. An additional extension step was performed for 10 min at 72°C. DNA sequencing was performed to confirm that all constructs had the desired sequence and open reading frame. All plasmids were transformed into DH5α-competent *E. coli*. Plasmid DNA copies were amplified in liquid culture and purified using a plasmid mini kit.

### Expression of recombinant fusion proteins

A single transformed BL21(DE3) colony was inoculated into 10 ml Luria-Bertani (LB) medium supplemented with ampicillin (100 g/ml) followed by agitation at 250 rpm overnight at 37°C. Then, 3 ml culture was transferred to 300 ml fresh LB medium in a 1,000 ml shake flask. The culture was grown at 37°C with 250 rpm agitation until the optical density at 600 nm (OD600) reached 1.2. Then, induction was performed with the addition of 0.3 ml 1 M isopropyl β-D-1-thiogalactopyranoside (IPTG). At 5 h after induction, a 1 ml sample was collected. The pellet was resuspended in 100 *μ*l ddH_2_O, mixed with 2X sodium dodecyl sulfate (SDS) loading buffer [0.0625 M Tris-HCl (pH 6.8), 2% SDS, 25% glycerol, 5%-mercaptoethanol and 0.01% bromphenol blue] and heated at 95°C for 4 min. Following centrifugation at 10,000 × g for 5 min, 20 *μ*l supernatant was analyzed by SDS-polyacrylamide gel electrophoresis (PAGE) and stained by Coomassie blue R-250.

### Lysis of cells and purification of the recombinant fusion proteins

Following induction for 5 h, cells were harvested by centrifugation at 10,000 × g for 15 min from 4,000 ml culture, resuspended in 100 ml 20 mM lysis buffer (20 mM NaH_2_PO_4_, 0.5 mM NaCl and 10 mM imidazole; pH 7.4) and lysed with a high pressure homogenizer (pressure, 900 bar; one time). The supernatant collected following centrifugation at 15,000 × g for 20 min was loaded in a nickel-nitrilotriacetic acid (Ni-NTA) resin column pre-equilibrated with 20 mM lysis buffer. The column was washed with equilibration buffer and when the absorbance at 280 nm was <0.01, the column was eluted using 50 mM imidazole in 20 mM equilibration buffer followed by a linear gradient of 50–500 mM imidazole in 20 mM equilibration buffer. The ammonium sulfate powder was added to the above elution liquid containing the eluted target protein to a concentration of 1 M. The above elution was loaded in to the Phenyl-Sepharose Fast Flow (FF) column pre-equilibrated with 20 mM phosphate-buffered saline (PBS; pH 7.4) supplemented with 1 M ammonium sulfate. The column was washed with equilibration buffer and when the absorbance at 280 nm was below 0.01, the column was eluted using a linear gradient of 1-0 M ammonium sulfate in 20 mM phosphate buffer (pH 7.4). The elution peak from the Phenyl-Sepharose FF column was loaded in to the HiLoad™ 26/60 Superdex 75 and washed with 20 mM phosphate buffer (pH 7.4); then the target protein peak was collected. The purified recombinant proteins were collected and analyzed by SDS-PAGE analysis. The final purity of products was determined by means of size exclusion-high-performance liquid chromatography (SE-HPLC).

### SDS-PAGE analysis

SDS-PAGE was performed using 12% resolution gel on the MiniProtean 3 system (Bio-Rad, Hercules, CA, USA). Briefly, the whole *E. coli* cell lysate was loaded on the gel. Then, the gel was electrophoresed at 150 V for 1 h and stained with Coomassie brilliant blue R-250. Low molecular weight protein markers were rabbit phosphorylase B (97.2 kDa), bovine serum albumin (66.4 kDa), rabbit actin (44.3 kDa), bovine carbonic anhydrase (29.0 kDa), soybean trypsin inhibitor (20.1 kDa) and hen egg white lysozyme (14.3 kDa).

### Identification of recombinant fusion proteins with western blotting

Following electrophoresis at 150 V for 1 h, the target protein was transferred to a polyvinylidene difluoride (PVDF) membrane using a Bio-Rad semidry apparatus with transfer buffer [10 mM CAPS buffer (pH 11), 10% methanol]. The PVDF membrane, wetted in 100% methanol, was soaked in transfer buffer before being placed on the PAGE gel. After being coated with anti-PSCA monoclonal antibody, the membrane was incubated at 4°C for 24 h. Then, it was washed and blocked with blocking buffer [3% bovine serum albumin V in Tris-buffered saline with 0.05% Tween-20 (TBST); pH 7.2]. Following three TBST washes for 5 min, the membrane was incubated for 1 h at 37°C with FITC-conjugated goat anti-rabbit IgG antibody followed by coloration with 3,3-diaminobenzidine (DAB) tetrahydrochlorate using a DAB kit according to the manufacturer’s instructions.

## Results

### Construction of expression plasmids

The PSCA gene and various structural domains of HSP70 were amplified by PCR with the respective primers. Then, the PSCA was cloned into pET21a(+) with the amino-terminus, carboxyl-terminus and overall length of HSP70, by enzyme digestion to construct the recombinant plasmids pET21-PSCA-HSPN, pET21-PSCA-HSPC and pET21-PSCA-HSP, respectively. The recombinant plasmids containing the target gene sequences were further analyzed by restriction enzymatic pattern and finally confirmed to be in full accord with sequences issued by GenBank [PSCA cDNA (GenBank original accession no. AF04F3498) and HSP70 cDNA (no. NM005345)].

### Expression of the recombinant fusion proteins

**Figure 1:**
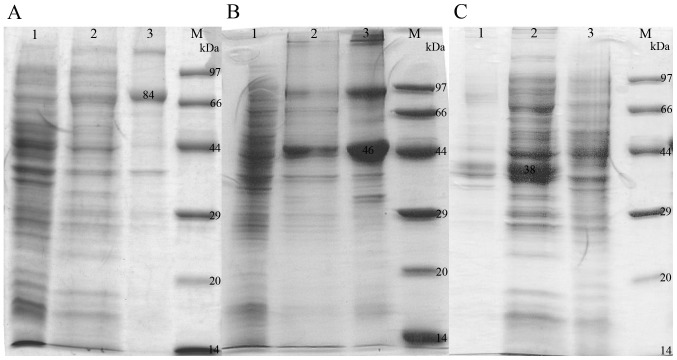
SDS-PAGE analysis of recombinant fusion proteins. (A) Lane 1, uninduced BL21(DE3)/pET21-PSCA-HSP; lane 2, induced BL21(DE3)/pET21-PSCAHSP, supernatant of the bacterial lysate; lane 3, induced BL21(DE3)/pET21-PSCA-HSP, pellet of the bacterial lysate; lane M, low molecular protein marker (97.2, 66.4, 44.3, 29.0, 20.1 and 14.3 kDa). (B) Lane 1, uninduced BL21(DE3)/pET21-PSCA-HSPN; lane 2, induced BL21(DE3)/ pET21-PSCA-HSPN, super-natant of the bacterial lysate; lane 3, induced BL21 (DE3)/pET21-PSCA-HSPN, pellet of the bacterial lysate; lane M, low molecular protein marker (97.2, 66.4, 44.3, 29.0, 20.1 and 14.3 kDa). (C) Lane 1, induced BL21(DE3)/pET21-PSCA-HSPC, pellet of the bacterial lysate; lane 2, induced BL21(DE3)/pET21-PSCA-HSPC, supernatant of the bacterial lysate; lane 3, uninduced BL21(DE3)/pET21-PSCA-HSPC; lane M, low molecular protein marker (97.2, 66.4, 44.3, 29.0, 20.1 and 14.3 kDa). SDS-PAGE, sodium dodecyl sulfate-polyacrylamide gel electrophoresis; PSCA, prostate stem cell antigen; HSP, heat shock protein.

When the OD600 of the culture reached 1.2, *E. coli* cells harboring expression plasmids were treated with IPTG at a final concentration of 1 mM. The expression of proteins corresponding to the predicted size were induced in the presence of IPTG. The proteins PSCA-HSPC and PSCA-HSP expressed in *E. coli* were confirmed to exist in soluble form by SDS-PAGE analysis (Fig. 1).

### Purification and identification of recombinant fusion proteins

**Figure 2:**
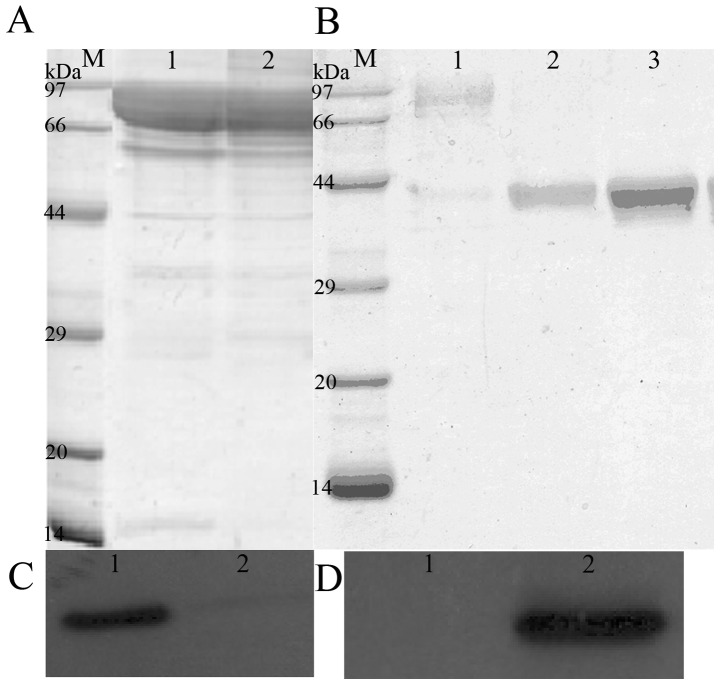
Purification and identification of recombinant fusion proteins. SDS-PAGE analysis of (A) PSCA-HSP and (B) SDS-PAGE analysis of PSCA-HSPC following purification with Ni-NTA resin, Phenyl-Sephrose Fast Flow and Superdex 75. (C) Identification of PSCA-HSP with western blotting. Lane 1, induced BL21(DE3)/pET21-PSCA-HSP; lane 2, uninduced BL21(DE3)/pET21-PSCA-HSP. (D) Identification of PSCA-HSPC with western blotting. Lane 1, uninduced BL21(DE3)/pET21-PSCA-HSPC; lane 2, induced BL21(DE3)/pET21-PSCA-HSPC. SDS-PAGE, sodium dodecyl sulfate-polyacrylamide gel electrophoresis; PSCA, prostate stem cell antigen; HSP, heat shock protein; Ni-NTA, nickel-nitrilotriacetic acid.

The cell paste was collected following induction. Recombinant PSCA-HSPC (Fig. 2A) and PSCA-HSP (Fig. 2B) were purified from the supernatant of the bacteria lysate following treatment with a combination of Ni-NTA resin, Phenyl-Sepharose FF and HiLoad™ 26/60 Superdex 75. A purity up to 95% was achieved, as confirmed by SDS-PAGE.

### Identification of recombinant fusion proteins with western blotting

After the proteins were transferred to the PVDF membranes, the PVDF membranes were treated successively with anti-PSCA monoclonal antibody and FITC-conjugated goat anti-rabbit IgG antibody. Western blotting confirmed the presence of PSCA (Fig. 2C and D).

## Discussion

The PSCA gene encodes a 123 amino acid protein. Studies have identified a low-expression of the protein in normal prostate and a high-expression in prostate, bladder and pancreatic cancer (10,11). Further studies demonstrated that the antibody of PSCA inhibits the growth of prostate cancer (12,13). However, is is difficult to express PSCA in a soluble condition in *E. coli* due to its complicated spatial structure, which makes it difficult to develop the vaccine for prostate cancer treatment. Therefore, a method to enhance the soluble expression of PSCA and elevate the immunogenicity of PSCA must be established. If assisted with a suitable adjuvant molecule, the immunogenicity and solubility of PSCA may be enhanced, which may improve the potency of protein vaccines based on PSCA.

The function of HSP70 in immunoadjuvant therapy has been identified and a number of studies have confirmed that vaccination with HSP70-peptide complexes and HSP70-antigen fusion proteins reconstituted *in vitro* with genetic recombination elicit antitumor immune responses (14–17). In addition, according to the results of our pre-experiment, HSP70 increases the potency of a DNA vaccine based on PSCA with strong cellular and humoral immune responses, which inhibit the growth of PSCA-expressing tumors and prolong the survival time of vaccinated mice (18). Moreover, coupling antigens to the amino-terminus of HSP70 may induce stronger immune responses than coupling to the carboxyl-terminus (18). HSP70 has two functional domains: the carboxyl-terminus is a peptide-binding domain, which provokes the production of cytokines and the amino-terminus is the ATPase domain, which is not capable of provoking the generation of cytokines. This terminal inhibits the secretion of interleukin (IL)-10 and transforming growth factor (TGF)-β (19). The antibody titres of anti-HSP70 or anti-HSPC generated by HSPC are lower compared with those generated by HSP70 (20).

In the present study, we presented evidence that human HSP70 enhances the solubility of PSCA. PSCA-HSPC and PSCA-HSP were successfully expressed to a high level in *E. coli* in soluble form and it is convenient for purification. PSCA-HSPN existed in insoluble form. Following three steps of purification, a purity of greater than 95% of the recombinant fusion proteins was obtained. Western blotting revealed that the PSCA and recombinant fusion proteins obtained via purification had the same immunological characteristics.

In conclusion, the present study confirmed the potency of human HSP70 as a molecular chaperone for a recombinant protein vaccine, which lays the foundation for the development of vaccines for prostate cancer and further clinical research.
